# Improving Patient Health Literacy During Telehealth Visits Through Remote Teach-Back Methods Training for Family Medicine Residents: Pilot 2-Arm Cluster, Nonrandomized Controlled Trial

**DOI:** 10.2196/51541

**Published:** 2023-11-16

**Authors:** Shanikque Barksdale, Shannon Stark Taylor, Shaniece Criss, Karen Kemper, Daniela B. Friedman, Wanda Thompson, Lorie Donelle, Phyllis MacGilvray, Nabil Natafgi

**Affiliations:** 1 Department of Health Services Policy and Management Arnold School of Public Health University of South Carolina Columbia, SC United States; 2 School of Medicine Greenville University of South Carolina Greenville, SC United States; 3 Family Medicine Residency Greenville Prisma Health Greenville, SC United States; 4 Department of Health Sciences Furman University Greenville, SC United States; 5 Department of Public Health Sciences College of Behavioral, Social and Health Sciences Clemson University Clemson, SC United States; 6 Department of Health Promotion, Education, and Behavior Arnold School of Public Health University of South Carolina Columbia, SC United States; 7 Patient Engagement Studio University of South Carolina Greenville, SC United States; 8 College of Nursing University of South Carolina Columbia, SC United States; 9 Family Medicine Prisma Health Greenville, SC United States

**Keywords:** digital health, family medicine, health literacy, medical residents, patient engagement, patient-centered, residency program, teach-back, telehealth, telemedicine, virtual care

## Abstract

**Background:**

As telemedicine plays an increasing role in health care delivery, providers are expected to receive adequate training to effectively communicate with patients during telemedicine encounters. Teach-back is an approach that verifies patients’ understanding of the health care information provided by health care professionals. Including patients in the design and development of teach-back training content for providers can result in more relevant training content. However, only a limited number of studies embrace patient engagement in this capacity, and none for remote care settings.

**Objective:**

We aimed to design and evaluate the feasibility of patient-centered, telehealth-focused teach-back training for family medicine residents to promote the use of teach-back during remote visits.

**Methods:**

We codeveloped the POTENTIAL (Platform to Enhance Teach-Back Methods in Virtual Care Visits) curriculum for medical residents to promote teach-back during remote visits. A patient participated in the development of the workshop’s videos and in a patient-provider panel about teach-back. We conducted a pilot, 2-arm cluster, nonrandomized controlled trial. Family medicine residents at the intervention site (n=12) received didactic and simulation-based training in addition to weekly cues-to-action. Assessment included pre- and postsurveys, observations of residents, and interviews with patients and providers. To assess differences between pre- and postintervention scores among the intervention group, chi-square and 1-tailed *t* tests were used. A total of 4 difference-in-difference models were constructed to evaluate prepost differences between intervention and control groups for each of the following outcomes: familiarity with teach-back, importance of teach-back, confidence in teach-back ability, and ease of use of teach-back.

**Results:**

Medical residents highly rated their experience of the teach-back training sessions (mean 8.6/10). Most residents (9/12, 75%) used plain language during training simulations, and over half asked the role-playing patient to use their own words to explain what they were told during the encounter. Postintervention, there was an increase in residents’ confidence in their ability to use teach-back (mean 7.33 vs 7.83; *P*=.04), but there was no statistically significant difference in familiarity with, perception of importance, or ease of use of teach-back. None of the difference-in-difference models were statistically significant. The main barrier to practicing teach-back was time constraints.

**Conclusions:**

This study highlights ways to effectively integrate best-practice training in telehealth teach-back skills into a medical residency program. At the same time, this pilot study points to important opportunities for improvement for similar interventions in future larger-scale implementation efforts, as well as ways to mitigate providers’ concerns or barriers to incorporating teach-back in their practice. Teach-back can impact remote practice by increasing providers’ ability to actively engage and empower patients by using the features (whiteboards, chat rooms, and mini-views) of their remote platform.

## Introduction

Telemedicine has become an increasingly used communication channel for patients, especially for the management of chronic conditions [1,2]. This has significant implications for the care delivery process, including the potential disruption of provider-patient communication and the confirmation of patient understanding of their diagnosis, treatment, and disease management plan. Furthermore, for most medical trainees, the pandemic was their first experience conducting telemedicine visits, and few had received any related training [3-6].

Health literacy, defined as “the ability to obtain, process, and understand basic health information and services to make appropriate health decisions,” is a key element to achieving health equity [7,8]. Nationally, only 12% of adults have proficient health literacy skills, resulting in difficulty with or an inability to read and understand medical information [7,9]. This can lead to feelings of shame and stigma, a lack of meaningful patient-provider communication, and a decrease in treatment adherence [10-13]. Notably, Healthy People 2030 extended its focus on health literacy to include organizational literacy, which highlights the role that health-related organizations can play in improving health literacy [14-16]. Their role includes implementing health literacy strategies at the system level to improve patient-provider interactions and health outcomes [14-16].

A key element among these strategies is the use of the teach-back method [17-19]. The teach-back method is an approach that verifies patients’ understanding of the health care information provided by health care professionals by asking them to reiterate, in their own words, the key information they need to know about their health conditions and the treatments they have been prescribed [19-23]. Research has shown that the use of the teach-back approach has a positive effect on the health outcomes of patients, including adherence to medication, quality of life, rehabilitation, patients’ overall care plans, and satisfaction with health care provider interactions [17,24-30].

Despite these benefits, health care providers frequently neglect to implement teach-back into patient-provider interactions [20,27,31]. This oversight is often attributed to concerns about time constraints, lack of training, and fear that patients will be offended by the providers’ attempt to use the teach-back approach [17,27]. To address this speculation, Anderson and colleagues [32] developed the 5Ts (Triage, Tools, Take Responsibility, Tell Me, and Try Again) for Teach Back. These concepts focus on the effective delivery of information and the evaluation of a patient’s comprehension of the messages conveyed by the physician [32]. The implementation of the 5Ts into practice ensures that the provider is focusing on the most important health care topics for the patient while also making sure that the patient understands that the request to reiterate the information to the provider is a way for the provider to confirm there is a shared understanding among the provider and the patient of the messages conveyed in the health care encounter [32].

Telehealth literacy is “a combination of elements of technologic and health literacy that allows for a patient to access, enable, and navigate their telehealth platform” [33]. As telemedicine plays an increasing role in health care delivery, it is imperative that providers receive adequate training to communicate with patients effectively during telemedicine encounters [3-6,34]. It is critical to support the development and use of providers’ skills to effectively communicate with patients during telemedicine appointments to optimize health outcomes, specifically in relation to the use of teach-back methods. Telehealth literacy requires specific communication skills that, to our knowledge, are not currently part of medical student and resident training or institutional continuing education.

In the context of telehealth, specific communication skills include (1) remote presence and engagement, where providers need to establish a remote presence that conveys empathy and trust despite the physical distance. Techniques for building rapport in a remote environment can include maintaining eye contact through the camera, using verbal cues to express empathy, and ensuring a comfortable and nondistracting background; (2) technological proficiency, as health care professionals must be proficient in the use of telehealth platforms and associated tools, such as remote whiteboards, chat rooms, and screen sharing. They need to guide patients through these platforms and troubleshoot technical issues to ensure effective communication; (3) effective use of visual aids, as telehealth often relies on visual aids and digital resources, providers should be skilled in using these aids to enhance patient understanding, such as annotating diagrams or sharing educational materials electronically; (4) privacy and security considerations*,* where providers are expected to communicate how patient information will be safeguarded and address any concerns patients may have about the security of remote interactions; and (5) active listening and adaptation are particularly important in a telehealth setting, as providers need to rely more on verbal cues and active listening to assess patient understanding. This includes asking open-ended questions, summarizing key points, and adapting their communication style based on patient needs.

There is evidence that various training methods are effective in promoting and encouraging the use of teach-back; however, there is limited evidence regarding the effectiveness of various teaching techniques in preparing health care professionals to use teach-back within a telehealth setting through a health literacy lens [35]. Improving the health care provider’s ability to effectively communicate with patients and use teach-back methods during a remote visit can support patients’ understanding of treatment and diagnosis and improve their ability to find, understand, and apply health information and services regarding their chronic condition. Furthermore, including patients in the design and development of teach-back training content for providers can result in more relevant training content. However, only a limited number of studies embrace patient engagement in this capacity, and none for remote care settings [36]. Therefore, this study aims to implement a patient-centered teach-back intervention among medical residents and evaluate the feasibility and acceptability of residents implementing the teach-back approach during telehealth visits.

## Methods

### Study Design, Setting, and Population

**Figure 1:**
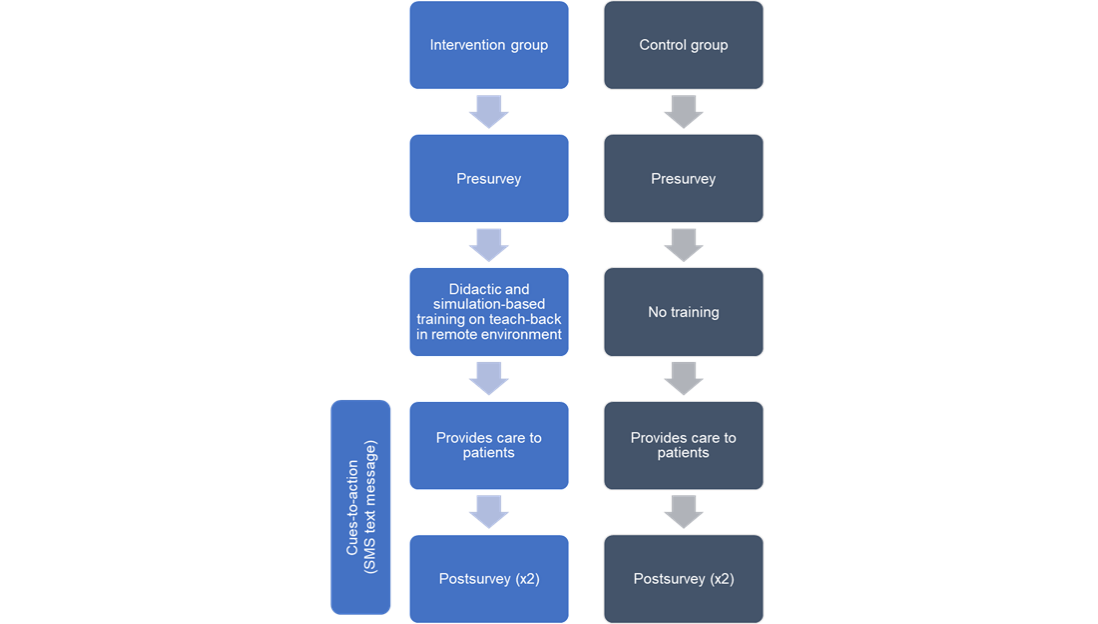
Schematic diagram of study design: 2-arm cluster, nonrandomized controlled trial.

We conducted a pilot study of a 2-arm cluster, nonrandomized controlled trial (Figure 1). The study was conducted in the Family Medicine Primary Care practice offices of one of the largest teaching health systems in South Carolina. A total of 3 family medicine and primary care residency clinics affiliated with the health system and located in South Carolina were allocated to either the intervention arm (1 clinic) or the control arm (2 clinics). Cluster allocation was chosen to minimize the interaction between the residents who receive the intervention and those who do not. Each clinic provides comprehensive primary care services for residents of the surrounding catchment area. The practice is recognized as a patient-centered medical home by the National Committee for Quality Assurance, which is a health care setting that emphasizes partnerships between patients, their providers, and their families, when appropriate.

One site with a total of 20 family medicine residents across the 3 years of postgraduate medical education serving an urban county of around 518,904 county inhabitants was allocated to the intervention arm. In the intervention arm, the medical residents (n=12; participation rate=60%) received didactic and simulation-based training on teach-back methods in a remote environment in addition to weekly cues-to-action. Additionally, the intervention residents participated in pre- and postsurveys, observations of residents, and interviews. A total of 2 other remote sites with a total of 24 family medicine residents across the 3 years of postgraduate medical education serving urban and suburban counties of around 415,067 inhabitants were allocated for the control arm. In the control arm, medical residents only participated in the pre- and postsurvey assessments (8/24, 33.3%, and 6/24, 25% response rate, respectively). The allocation of sites to intervention and control arms was based on the number of residents in each site to achieve a comparable number of residents in the control and intervention groups.

### POTENTIAL Program Development

The POTENTIAL (Platform to Enhance Teach-Back Methods in Virtual Care Visits) curriculum for medical residents was created to use teach-back to improve patient experiences during remote visits. The Adult Learning Theory provided the framework for the development of the curriculum. According to this theory, adults want to be involved in their learning experience and want to know how information relates to their past and current experiences [37,38]. To accomplish this goal, the program focused on using panel discussions, simulations, lectures, and videos.

#### Patient Involvement

The University of South Carolina’s Patient Engagement Studio (PES) was involved in the development and implementation of the POTENTIAL Program. The PES brings together patients and caregivers, community groups, health system innovators and clinicians, and academic researchers to produce meaningful research and innovation that advances health and research outcomes [39]. The Remote Patient panel of the PES—which includes a panel of patient experts with lived experience of chronic conditions who used telehealth—were involved in the development and review of the POTENTIAL curriculum and provided feedback on its content and ability to convey the importance of teach-back in patient-provider communication [40]. Additionally, a patient expert (one of the coauthors) participated in the development of the workshop’s videos and in a patient-provider panel about teach-back.

#### Curriculum

The intervention group attended a 1-day training session that consisted of two 90-minute workshops (Table 1). These workshops aimed to meet nine learning outcomes: (1) compare and contrast provider and patient experience during a Remote patient encounter, (2) describe principles of health literacy, (3) list the impact of limited health literacy on patient outcomes, (4) describe the 4 elements addressed in patient education, (5) evaluate patients relative to the patient activation continuum, (6) describe the role of teaching patients problem-solving skills to manage chronic conditions, (7) list the 5Ts of the teach-back method, (8) describe best practices for using teach-back during remote patient encounters, and (9) demonstrate competence using the teach-back method during a remote patient encounter.

**Table 1:** Overview of the components of the “teach-back during remote visits” training for family medicine graduate medical residents at a teaching medical center in South Carolina.

Session	Learning objectives	Education method	Assessment
Review of Health Literacy and Teach-Back	Compare and contrast a provider and patient experience during a remote patient encounter. Describe principles of health literacy. List the impact of poor health literacy on patient outcomes. Describe the 4 elements addressed in patient activation. Evaluate patients relative to the patient activation continuum. Describe the role of teaching patients problem-solving skills to manage chronic conditions.	Lecture Discussions Videos Role play	Observation
Teach-Back in Remote Settings and Simulations	List the 5Ts^a^ of the Teach-Back method. Describe best practices for using Teach-Back during remote patient encounters. Demonstrate competence using the Teach-Back method during a remote patient encounter.	Lecture Teach-back simulation Panel discussion Reflections	Surveys Teach-back observation tool

^a^5Ts: Triage, Tools, Take Responsibility, Tell Me, and Try Again.

To assist residents in accomplishing these learning outcomes, the first session, “Review of Health Literacy and Teach-Back” consisted of an icebreaker, a review of health literacy, an explanation of teach-back, and a demonstration of how to use teach-back in the clinical setting. This session used videos, role-play, and presentation to explain and demonstrate the different scenarios in which teach-back would benefit patients and providers. The second session, “Teach-Back in Remote Settings and Simulations,” emphasized the use of teach-back in a remote setting. To accomplish this, residents were exposed to different strategies to use in a remote setting to maximize the impact of teach-back. These strategies highlighted ways to use remote features such as mini view, screenshare, whiteboard, and in-call chat. Additionally, the residents were also introduced to environmental (ie, favorable lighting and limiting distracting clutter) and behavioral (ie, intentional movement and narrated behavior) best practices that support the implementation of teach-back in a remote care setting. Following exposure to the various teach-back methods to use in a remote setting, the residents were allotted time to practice using teach-back during remote simulations. The simulations were followed by a panel discussion with a provider who uses teach-back and a patient who has experienced teach-back. This provided the residents with the opportunity to hear and learn from those with first-hand experiences of teach-back being implemented in the clinical setting. At the end of the panel, the residents were provided resources and the opportunity to offer reflections regarding the information they learned that day. The sessions were moderated by the director of the Graduate Medical Education program and provided by 2 doctoral-trained health literacy experts, a physician, and a patient.

Residents also received weekly cues to action by SMS text message 3 months post training (Table 2). The weekly cues to action were developed using a staged social cognitive approach to message design outlined in Maibach and Cotton’s work in “Moving People to Behavior Change” [41]. This approach integrates social cognitive theory and the transtheoretical model to guide the development of messages tailored to influence specific cognitive factors at each stage of change. For example, messages designed to promote movement from preparation to action stages targeted outcome expectations, self-efficacy, and skills related to the use of teach-back during remote patient visits. Positive outcome expectations were promoted through messages of social reinforcement of the behavior. Self-efficacy was promoted through messages that provided information about the 5 key teach-back strategies and messages that encouraged taking small steps to integrate teach-back strategies into remote patient visits. Messages targeted behavior transition from preparation to action and action to maintenance. For each behavior transition targeted, the social cognitive factors of knowledge, outcome expectations, self-efficacy, skills, and personal goals were addressed. A total of 29 messages were developed using the staged social cognitive approach to serve as reminders of the different techniques that the residents could use to effectively use teach-back and ensure that the patient understands the care plan that has been created for them (Table 2).

**Table 2:** Examples of weekly cues to action sent to family medicine graduate medical residents at a teaching medical center in South Carolina.

Stage of change	Theoretical targets	Message objective	Message
N/A^a^	N/A	Introductory text	Hi! Thank you for taking part in the POTENTIAL Project to improve teach-back during virtual visits. We’ll send you weekly text reminders to use teach-back in your clinic. If you have questions or concerns, plz text XXX or XXX. The POTENTIAL Team.
Preparation to action	Self-efficacy to try using teach-back during remote visits	To demonstrate that the use of teach-back won’t hinder providers ability to stick to time constraints of visits	Did you know teach-back only adds a minute or two to a patient visit when done properly?
Preparation to action	Self-efficacy to try using teach-back during remote visits Building Teach Back skills	To demonstrate the use or understanding of small steps to implement teach-back into practice To understand how to use the 5Ts^b^ of teach-back	Don’t forget the 5Ts for Teach-back! The first one is Triage. Focus on one topic for teach back during today’s patient encounter
Action to maintenance	Skills	To demonstrate the implementation of teach-back and the use of remote tools	When in a virtual care visit be sure to use the chat, share screen, and whiteboard features! Interactive visits can help keep patient engaged!
Action to maintenance	Self-efficacy Outcome expectations	To encourage self-evaluation to overcome setbacks during teach-back encounters To encourage modification of teach-back practice until desired comfort level with the technique is achieved and maintained	Practice makes perfect! With a little time and frequent use, teach-back will feel less awkward and have no impact on the length of a patient visit.

^a^Not applicable.

^b^5Ts: Triage, Tools, Take Responsibility, Tell Me, and Try Again.

### Evaluation

The overall assessment of the POTENTIAL program followed a 4-level assessment geared toward the intervention group. The assessment included (1) course evaluation, (2) observations of residents during simulation exercises, (3) pre- and postsurveys, and (4) interviews with patients and residents. The control group was assessed only through surveys that were administered, while the pre- and postsurveys were administered to the intervention group.

#### Course Evaluation

To evaluate the curriculum’s implementation, medical residents were asked to complete a course evaluation, asking for feedback on the content and opportunities for improvement. The course evaluation was completed anonymously on the web at the end of the sessions. The evaluation asked residents to rate their experience on a scale of 1 (poor) to 10 (excellent). Residents were asked which topic they liked best from each of the sessions and if they felt they learned something new. These questions were followed by open-ended questions about what residents liked best about the sessions and opportunities for improvement.

#### Simulation Practice Observations

During session 2, the residents were given the opportunity to practice teach-back with remote patient stimulations using VidyoConnect (Vidyo), the remote visit platform used at the health system. The simulations were carried out at the University of South Carolina School of Medicine Simulation Center. For this exercise, each resident was assigned to one of 2 roles: patient or provider. They were given different patient case scenarios and tasked with providing care for the patient using teach-back. The practice sessions and content of the interactions were recorded and observed by one of the researchers. Each resident’s practice session was reviewed and scored using a Teach-Back Observation Tool adopted from the Always Use Teach-Back training toolkit [42].

#### Pre- and Postsurveys

Residents also completed pre- and postintervention surveys. The pre- and postsurveys were given to the intervention and control groups. The surveys measured the medical residents’ perceptions of their teach-back use through a modified version of the “Always Use Teach-Back Confidence and Conviction Scale.” Surveys were collected before (at baseline before the training session) and after the implementation of the training.

#### Qualitative Data

As a supplemental component, patient and resident volunteers were recruited to provide information on their experiences with teach-back postintervention. A total of 3 patient interviews and 1 resident interview were conducted remotely by research team members. In addition, the surveys included open-ended questions to assess experiences and barriers or facilitators of teach-back during remote care visits.

### Data Analysis

For the pilot study, descriptive statistics, specifically frequencies, proportions, percentages, means, and SD, were reported. To assess differences between pre- and postintervention scores among the intervention group, chi-square testing for categorical variables and a 1-tailed *t* test for continuous variables were used. A total of 4 difference-in-difference models were constructed to evaluate prepost differences between intervention and control groups for each of the 4 continuous outcomes (familiarity with teach-back, importance of teach-back, confidence in teach-back ability, and ease of use of teach-back). Given our aims and the fact that the numbers in this pilot study were too small for reliable inferences, between-group comparisons of outcomes were limited. All quantitative analyses were conducted using Stata 16.1 (StataCorp). The qualitative data (interviews and open-ended survey questions) were thematically analyzed. This manuscript adheres to CONSORT (Consolidated Standards of Reporting Trials) guidelines.

### Ethical Considerations

This study received ethical approval from the Prisma Health Institutional Review Board (Pro00110006). The intervention was provided to family medicine residents as a part of their graduate medical education program. For supplemental interviews, participants were provided a brief description of the study and given the opportunity to ask any questions. If the individual agreed to participate in the study, oral informed consent was obtained. All study records were deidentified and kept confidential. Each interviewee received a US $20 gift card at the conclusion of their in-depth interview.

## Results

### Overview

Of the 20 family medicine residents at the intervention site, 12 (60%) attended the training sessions. Two-thirds (64.9%) of the residents in the study identified as women. One-half (48.6%) of the residents were in their second year of postgraduate medical education, and 27% (10/37) were in their third year of the program.

### Course Evaluation

Residents rated their teach-back training session experience at an average of 8.64 (mean 9). From each of the 2 sessions, their favorite parts were the discussion on health literacy (7/11, 64% of residents) and the panel discussion (5/11, 46%). Residents reported that they enjoyed the panel because it provided the patient advocate’s perspective and allowed them to hear the experience of a provider who actively performs teach-back in their practice. Almost all (100%) of the residents stated that they learned something new from the teach-back in a remote care setting workshop. Regarding opportunities for improvement, residents would like to learn more about how to use the telemedicine platform to maximize patient engagement and to receive clearer instructions on how to navigate the simulation demonstration. Furthermore, there was a suggestion to focus more on the tools and phrases that can be used to promote teach-back during patient encounters.

### Simulations

During the practice simulation, approximately 50% (6/12) of residents in the provider role used the mini-view to see their patient and make eye contact. Additionally, 75% (9/12) of the residents used plain language while speaking to the patient. Similarly, 75% (9/12) asked the patient to use their own words to explain what they were told during the encounter. It was also noted that many residents still tended to ask the patient “yes” or “no” questions.

### Residents Surveys

When asked about their communication practices in remote care visits that happened in the previous month, most of the residents, preintervention, indicated that during patient encounters they used open-ended questions (11/12, 91.67%) and plain (patient-friendly) language (11/12, 91.67%; Table 3). Post intervention, 100% and 87.5% (7/8) of residents stated that they used plain language and open-ended questions, respectively. Overall, the least commonly used practices were asking the patient to explain, in their own words, what they were told (0/8, 0%), explaining and checking again if the patient is unable to teach-back (1/8, 27%), and documenting the use of and patients’ response to teach-back (1/8, 16%).

**Table 3:** Communication and other teach-back-related practices (more than 50% of clinical remote visits) for medical residents during remote care visits among those who conducted a remote care visit in the past month, intervention site.

Communication and other practices^a^	Presurvery (N=12), n (%)	Postsurvey (N=8)^b^, n (%)
Display comfortable body language, make eye contact.	10 (83)	8 (100)
Use plain (patient-friendly) language (ie, avoid jargon).	11 (92)	8 (100)
Use a caring tone of voice and attitude.	10 (83)	6 (75)
Use open-ended questions (ie, questions that cannot be answered with a yes or no).	11 (92)	7 (88)
Engage with the patient by keeping your camera running all the time.	8 (67)	8 (100)
Take responsibility for making sure you were clear.	8 (67)	3 (38)
Use reader-friendly print materials to support learning.	8 (67)	6 (75)
Include family members and caregivers if they were present.	7 (58)	6 (75)
Keeping mini view (to see patient nonverbal cues) all the time.	5 (42)	4 (50)
Ask the patient to explain, in their own words, what they were told.	5 (42)	0 (0)
Explain and check again if the patient is unable to teach back.	4 (33)	1 (13)
Document the use of and the patient’s response to teach-back.	2 (17)	1 (13)

^a^Residents can choose multiple responses.

^b^A total of 3 residents did not have any telemedicine encounter in the past month.

Table 4 summarizes the survey results, showing differences in pre- and postintervention scores for both intervention and control groups. While almost all of the residents believed that teach-back improves the quality of care for patients, only 58% (7/12) of the intervention residents and 25% (2/8) of the control residents believed that teach-back should be mandatory (preintervention). Interestingly, this finding flipped in the postintervention survey, where only 18% (2/11) of intervention residents felt it should be mandatory while 50% (3/6) of control residents felt it should be mandatory. The difference in pre- versus postintervention among the intervention group was only marginally significant, and the difference in the control group was not statistically significant.

**Table 4:** Family medicine graduate medical residents’ characteristics and perspectives on teach-back during remote visits, all sites (including both pre- and postintervention surveys), between October 2021 and April 2022.

Variable			Intervention site								Control site				
			Pre (n=12), n (%)		Post (n=11), n (%)		*P* value			Pre (n=8), n (%)			Post (n=6), n (%)	*P* value	
**Gender**								.48							.04
	Man	5 (42)		3 (27)					4 (50)			0 (0)			
	Woman	7 (58)		7 (64)					4 (50)			6 (100)			
**Year of residency**								.83							.39
	PGY^a^ 1	3 (25)		2 (18)					3 (38)			1 (17)			
	PGY 2	5 (42)		4 (36)					5 (63)			4 (67)			
	PGY 3	4 (33)		5 (46)					0 (0)			1 (17)			
**Frequency of teach-back during remote visits**								.68							.56
	Do not do it now, but plan in future	2 (17)		3 (27)					2 (25)			2 (33)			
	25% of all remote encounters	6 (50)		3 (27)					1 (12)			1 (17)			
	50% of all remote encounters	3 (25)		3 (27)					3 (38)			2 (33)			
	75% of all remote encounters	1 (8)		2 (18)					2 (25)			0 (0)			
	100% of all remote encounters	0 (0)		0 (0)					0 (0)			1 (17)			
**Teach-back improves quality of care**								.95							N/A^b^
	Maybe	1 (8)		1 (9)					0 (0)			0 (0)			
	Yes	11 (92)		10 (91)					8 (100)			6 (100)			
**Should teach back be mandatory?**								.049							.33
	No	5 (42)		9 (82)					6 (75)			3 (50)			
	Yes	7 (58)		2 (18)					2 (25)			3 (50)			
**Continuing education on teach-back?**								.28							.37
	No	2 (17)		4 (36)					1 (12)			0 (0)			
	Yes	10 (83)		7 (64)					7 (88)			6 (100)			
**Case scenario: least effective question to assess understanding**								.11							.46
	Correct (Do you have any questions?)	6 (50)		9 (82)					4 (50)			3 (50)			
	Incorrect (Other teach-back statements)	6 (50)		2 (18)					4 (50)			3 (50)			

^a^PGY: Postgraduate year.

^b^Not applicable.

While all 4 outcomes assessed (familiarity with teach-back, importance of teach-back, confidence in teach-back ability, and ease of use of teach-back) increased in the expected direction (pre- vs postintervention), only one (confidence in teach-back ability) was statistically significant (mean 7.3 vs 8.4; *P*=.04; Table 5). Interestingly, those results were also mirrored in the control group, where confidence in teach-back ability also increased in the control group (mean 7.8 vs 9.0; *P*=.05). None of the 4 difference-in-difference models were statistically significant in relation to the difference in residents’ ratings of familiarity with, importance of, confidence in, and ease of using teach-back (results not shown).

**Table 5:** Family medicine graduate medical residents’ perspectives on teach-back during remote visits, all sites (including both pre- and postintervention surveys), between October 2021 and April 2022.

Variable	Intervention site				Control site			
	Pre (n=12), mean SD	Post (n=11), mean (SD)	*P* value	Pre (n=8), mean (SD)		Post (n=6), mean (SD)	*P* value	
Familiar with “teach-back”	8.08 (1.62)	8.91 (1.22)	.09	9.00 (0.93)		9.17 (1.33)	.40	
Importance of “teach-back”	8.25 (1.71)	8.45 (1.37)	.38	9.13 (1.12)		9.33 (1.03)	.37	
Confidence in “teach-back” ability	7.33 (1.56)	8.36 (1.02)	.40	7.75 (1.04)		9.00 (1.54)	.05	
Ease of using “teach-back”	6.17 (2.25)	6.91 (1.22)	.17	7.00 (2.00)		7.83 (1.33)	.20	

### Qualitative Data

Patient interviewees revealed positive experiences with remote care visits. Patients felt that their providers did a good job of verifying that they understood the instructions that were provided during the remote care encounters. Despite their positive experiences with remote visits, the patients did have suggestions for improvement. Among those suggestions were having a provider who solely provided telehealth visits and ensuring that providers had a quiet space to conduct such visits. The resident interview highlighted that effective implementation of teach-back happens when teaching patients how to administer treatments such as inhalers. The interview also indicated that time and determining which teach-back strategy was best to use during the visit were barriers.

Table 6 summarizes the open-ended survey responses from medical residents regarding their experiences and barriers with using teach-back during remote visits. For those who believed that teach-back should be mandatory, their reason was because “patient education is key, it empowers patients.” While those who felt it should not be mandated described that:

Teach back can be limited by a patient’s engagement and their education which can make teach back take longer than time allowed in an encounter. It is an effective tool when used appropriately and should be employed when appropriate.

The most common barrier was time, followed by patient literacy and communication, and technical difficulties. Of note, the experiences and barriers were not vastly different in the pre- and postintervention assessments.

**Table 6:** Experiences and barriers for family medicine graduate medical residents using teach-back during remote visits.

Preintervention			Postintervention
**Experiences**			
	I think it’s really helpful, better for patients. Does take a while which is hard with limited slots	Generally positive when you have an engaged patient	
	It’s effective to ensure understanding. It can take a lot of time with those with low education or those with low engagement.	Generally positive when there is time	
	I feel it is positive as it allows me to get a baseline of what the patient understands	It almost always highlights areas of misunderstanding	
	Prolongs visit	Positive (because it) ensure(s) patient understands	
**Barriers**			
	Only barrier is if patient has a baseline mental status of confusion or dementia so cannot use it directly. This is where involving family members in the plan of care is important	If the patient is hard of hearing or has a cognitive-deficiency due to dementia etc.	
	Time limit, do not have enough time during an encounter	Time mostly	
	Communication or use of translator, poor health literacy	Patient literacy, internet connectivity issues	
	Language, educational level	Engagement of patient, understanding that teach back is not insulting, time, and interest in putting plan of care into place	

### Discussion and Conclusion

This pilot study aimed to test the feasibility and acceptability of implementing a telemedicine-focused teach-back training program among family medicine residents in a multisite teaching medical center. This study demonstrates the feasibility and acceptability of implementing a 3-hour multicomponent workshop to enhance providers’ knowledge and awareness of teach-back approaches that can be augmented by cue-to-action SMS text messages to encourage practice change. This is in line with other trainings that are often scheduled to last anywhere from 1 hour to a full day and incorporate a variety of techniques such as role play, video, lectures, and small groups to assist the providers in their learning of and comfort with teach-back [8,18,43,44].

## Discussion

### Overview

In the post–COVID-19 pandemic world, the rate of telemedicine visits is expected to continue to be higher than prepandemic rates. The proportion of primary care providers using telemedicine increased from 5.3% before the pandemic to 46.2% during the pandemic [45]. According to a recent American Medical Association (AMA) survey, more than two-thirds (69%) of physicians are interested in continuing to offer telehealth services to their patients [46]. Furthermore, a 2022 patient consumer survey showed that 76% of users who had a telehealth visit would like to continue using it in the future [47]. This has led to calls for incorporating telemedicine into family medicine training [48].

Post intervention, there was a notable increase in residents’ confidence in their ability to use teach-back. Although the increase was observed in both the intervention and control groups, the intervention’s impact on confidence suggests that the curriculum holds promise in fostering improved communication skills. Furthermore, among those who conducted a remote visit, more medical residents who received the intervention indicated that they followed communication and other teach-back-related practices (eg, maintain eye contact, use plain language, use open-ended questions, etc) compared with those who did not. This aligns with other research in nontelehealth settings in which training on teach-back increased health professional usage of this technique [17,19,20,26,32,43,44,49].

However, it is important to note that the pilot study did not show statistically significant changes in familiarity with teach-back, perception of its importance, or ease of use of teach-back. The lack of significance could be attributed to the pilot’s small sample size, which may have limited the study’s power to detect smaller effect sizes. Therefore, while not statistically significant, the trends observed in these measures are intriguing and warrant further exploration in larger-scale implementation efforts. The difference-in-difference models also did not yield statistically significant results, likely due to the study’s exploratory nature and limited statistical power. However, the insights gained from these models provide valuable guidance for refining and optimizing future interventions. The improvement seen in the control group postsurvey could be partially attributed to the testing effect of the presurvey (testing threat to validity) and the Hawthorne effect, which is a bias that occurs when behavioral change occurs when you know you are being studied [50]. In fact, a total of 8 studies identified that the Hawthorne effect was responsible for the improvements in the control group in surgical settings [51]. Additionally, the control-group residents may have felt more confident based on the survey providing the specific components of the teach-back method [50-52].

Furthermore**,** residents offered several barriers they perceived to influence their use of teach-back strategies during telehealth appointments. While this information was collected before and following the pilot intervention, barriers were similar both times. The main barrier reported by medical residents in incorporating teach-back into practice was time constraints, suggesting that efforts to integrate teach-back seamlessly into the telehealth workflow could enhance its adoption. Residents reflected that patients’ cognitive and hearing issues; language barriers; limited education; limited literacy and health literacy; and low engagement (responsiveness) of patients were all barriers to being able to use teach-back methods with their patients. These patient-focused barriers are shown to impact patient-provider communication and medical outcomes [53-56]. However, the written and verbal communication and practices of health care providers and health care organizations can also strongly influence the patient experience and health outcomes [13,56]. With the focus of Healthy People 2030 on organizational health literacy, stressing the clinical context and the providers’ and health care organizations’ roles in helping to improve patient health literacy and engagement, it will be important to address the provider and organizational barriers [14,15].

Lastly, the positive outcomes of this study highlight the opportunity for future research to implement training of teach-back in a remote care setting on a larger scale. The use of a larger site will increase the generalizability of the study and provide greater evidence to support the impact that widespread adoption of such a curriculum could have on the quality of remote care. There is also an opportunity for future research to evaluate the impact of “cues to action” on patient engagement and the implementation of teach-back strategies in a remote care setting. This study also highlights the need for future research to evaluate simulations before and after learning sessions to fully capture the rate of behavior change that a training curriculum can produce.

### Strengths and Limitations

Although the experimental design of this feasibility study strengthens the quality of the results, several limitations should be considered when interpreting the findings. First, the study population (and hence sample size) was small, and this lowers the power to detect statistically significant differences between groups. However, the study included the entire population of medical residents, and the intervention participation rate (60%) and survey response rates (67%-100%) were acceptable. Second, in addition to the small sample size, data were collected in 1 medical residency group within a single large health care system (albeit multisites), and this may limit the generalizability of results to a broader medical residency context and to other health care providers, including those with different specialties, using telemedicine for patient appointments. Furthermore, the study did not randomly assign participants to intervention versus control groups; however, residents were cluster randomized to groups based on clinic geographic location. This was done to minimize social interaction threats that could occur if intervention and control groups were comingled within geographic locations. Lastly, some of the outcome measures (eg, familiarity with teach-back, importance of teach-back, and confidence in teach-back ability) relied on self-reported measures, which might not always accurately reflect actual behavior or performance. For those measures, we used a modified version of the “Always Use Teach-Back Confidence and Conviction Scale,” which is a widely used assessment tool based on the stages of change and behavioral change. This tool was used to highlight whether the medical residents perceived belief, confidence, and familiarity with teach-back increased because of the POTENTIAL curriculum. In addition, we have used other assessments, including observations and interviews with both providers and patients. Future studies should also consider looking at medical records to note any potential changes in health behaviors or outcomes relevant to this intervention.

### Conclusions

This pilot study highlights ways to effectively integrate best-practice training in telehealth teach-back skills into a graduate medical residency program. Specifically, it represents a crucial step in understanding the potential impact of the POTENTIAL curriculum on telehealth teach-back skills among medical residents, pointing to important opportunities for improvement for similar interventions in future larger-scale implementation efforts as well as ways to mitigate providers’ concerns or barriers to incorporating teach-back in their practice. Teach-back can impact remote practice by increasing providers’ ability to actively engage and empower patients by using the features (whiteboards, chat rooms, and mini-views) of their remote platform. In conclusion, this pilot study sets the stage for future research in this critical area and underscores the potential of the POTENTIAL curriculum to improve telehealth communication practices.

### Practice Implications

To provide a true patient-centered care experience to patients, we need to provide purposeful telemedicine-focused teach-back and health literacy training to family medicine residents. Such training is key to ensuring patient understanding, enhancing the health literacy of patients, and improving patient experience and outcomes in telemedicine. However, this type of training requires an elaborate and intentional effort to engage patients and their caregivers in the development and implementation of this intervention [57].

Future training should emphasize the organization’s role in patient health literacy and involve leadership in such discussions and educational programming with the goal of striving to become a health-literate organization. In expanding the focus to include organizational health literacy, Healthy People 2030 is clearly acknowledging that patient health literacy can be improved further upstream by prioritizing improvements in patient-provider communication and identifying patients in need of additional resources and support, including translation services or referral to resources such as local literacy services. Further research is needed to determine whether the use of teach-back in a remote environment has a significant impact on patients’ health outcomes and patient-provider communication.

## Abbreviations

5Ts: Triage, Tools, Take Responsibility, Tell Me, and Try Again
AMA: American Medical Association
CONSORT: Consolidated Standards of Reporting Trials
PES: Patient Engagement Studio
POTENTIAL: Platform to Enhance Teach-Back Methods in Virtual Care Visits

## Data Availability

The data sets generated during or analyzed during this study are available from the corresponding author on reasonable request.
